# Rethinking the history of peptic ulcer disease and its relevance for network epistemology

**DOI:** 10.1007/s40656-021-00466-8

**Published:** 2021-11-04

**Authors:** Bartosz Michał Radomski, Dunja Šešelja, Kim Naumann

**Affiliations:** 1 Institute for Philosophy II, Ruhr University Bochum, Bochum, Germany; 2 Philosophy and Ethics Group, TU Eindhoven, Eindhoven, The Netherlands; 3 Munich Center for Mathematical Philosophy, LMU Munich, Munich, Germany

**Keywords:** Peptic ulcer disease, Eddy Palmer, Digital textual analysis, Network epistemology

## Abstract

The history of the research on peptic ulcer disease (PUD) is characterized by a premature abandonment of the bacterial hypothesis, which subsequently had its comeback, leading to the discovery of Helicobacter pylori—the major cause of the disease. In this paper we examine the received view on this case, according to which the primary reason for the abandonment of the bacterial hypothesis in the mid-twentieth century was a large-scale study by a prominent gastroenterologist Palmer, which suggested no bacteria could be found in the human stomach. To this end, we employ the method of digital textual analysis and study the literature on the etiology of PUD published in the decade prior to Palmer’s article. Our findings suggest that the bacterial hypothesis had already been abandoned before the publication of Palmer’s paper, which challenges the widely held view that his study played a crucial role in the development of this episode. In view of this result, we argue that the PUD case does not illustrate harmful effects of a high degree of information flow, as it has frequently been claimed in the literature on network epistemology. Moreover, we argue that alternative examples of harmful effects of a high degree of information flow may be hard to find in the history of science.

## Introduction

The early 20th century research on peptic ulcer disease (PUD) is often mentioned as an example of scientific inquiry ‘gone wrong’ (e.g. Thagard, 2000; Gilbert, 2000; Solomon, 2001; Zollman, 2010; Wray, 2010; Miller, 2013; Šešelja & Straßer, 2014; O’Connor, 2020). As most accounts of this case report, from the 19th century on there were two major rivaling hypotheses of the disease: the acidity hypothesis, according to which the disease is caused by an excessive acidity of the stomach, and the bacterial hypothesis, which stipulated bacteria as the primary cause of the disease. In the mid-20th century the bacterial hypothesis was abandoned, and the research on PUD proceeded along the lines of the acidity research program. The latter focused on the study of various treatments aimed at achieving a chemical balance in the stomach, from antacid medications to surgical procedures, rather than on the identification of bacteria and their eradication. For three decades the research on PUD was based on a worse of the two hypotheses. It was only in the 1980s that Robin Warren and Barry Marshall discovered Helicobacter pylori, a bacterium which turned out to be the major cause of PUD. This discovery, for which Warren and Marshall received a Nobel Prize in Physiology or Medicine, led to the revival of the bacterial research program.

According to the received view on the history of this episode (originating primarily in Warren and Marshall, 1983; Marshall, 2002),^1^ the main reason for the abandonment of the bacterial hypothesis was a large-scale study by a prominent gastroenterologist, Palmer (1954). Palmer examined 1,180 subjects, fifth of whom were healthy individuals, while the remainder of the group were patients with gastrointestinal complaints. The study showed no presence of bacteria in the gastric mucosa of the subjects. As scientists Fukuda et al. (2002), reflecting on the history of this case, write:

[T]he hypothesis that PUD was caused by bacteria in the mucosa of the human stomach was rejected in 1954 by the major authority in American gastroenterology, [Palmer, 1954] despite consistent information in the preceding 50 years of bacteria that adhered to the gastric musosa $\dots$ His words ensured that the development of bacteriology in gastroenterology would be closed to the world as if frozen in ice. (p. 17)

His study established the dogma that bacteria could not live in the human stomach, and as a result, investigation of gastric bacteria attracted little attention for the next 20 years. (p. 20)

Nowadays we know that Palmer’s study was deeply misleading as it was based on a method unsuitable for detecting spiral bacteria (see Fukuda et al., 2002). As a result, this historical episode has become one of the central examples of an inquiry in which everything was done by the book in the sense that each individual scientist had good reasons to abandon the bacterial hypothesis, and yet, the scientific community on the whole was sidetracked towards a false theory for a long period of time (Zollman, 2010; Kummerfeld and Zollman, 2016; O’Connor, 2020). As such, the PUD case appears to be a nice example of the individual and group rationality coming apart in the sense that rational choices by individual scientists do not sum up to an optimal inquiry at the level of the given community. It also appears to be illustrative of how a wide dissemination of erroneous findings can sidetrack the entire scientific community.

However, such an interpretation of the events leaves some questions open. For instance, if Palmer’s study was that influential, how come nobody in the scientific community noticed potential problems with it? After all, warnings about the unsuitability of the method of staining used in Palmer’s study had previously been pointed out by Freedberg and Barron (1940), whom Palmer even cited in his paper. This is all the more surprising if we agree with Šešelja and Straßer (2014) that the bacterial research program (in spite of Palmer’s findings) had promising lines of inquiry in the 1950s, when it was largely abandoned. What is more, the alleged impact of Palmer’s study has never been corroborated by adequate historical evidence. Nor has the widely adopted view of the popularity of the bacterial hypothesis prior to Palmer’s publication ever received a proper evidential support.

In this paper we aim to advance this debate by conducting a critical examination of the received view on PUD, according to which, the main reason for the abandonment of the bacterial hypothesis was Palmer’s study. If the received narrative is correct, the abandonment of the bacterial hypothesis can be ascribed, for instance, to Palmer’s influence, which swayed the entire medical community. As Zollman (2010) writes: “It was the widespread acceptance of Palmer’s result which led to the premature abandonment of the diversity in scientific effort present a few years earlier.” (p. 21).

However, claims about Palmer’s influence are often asserted without considering the state of the research landscape prior to 1954. Our aim is therefore to examine whether the bacterial hypothesis of PUD had been largely abandoned already before Palmer’s publication. In order to uncover the details of this episode, we have used the method of digital textual analysis applied to the corpus of the English-language literature on PUD published in the decade prior to Palmer’s study. The reason why this point is especially interesting is that, if confirmed, it would have important repercussions for philosophical discussions of this episode.

First of all, the relevance of Palmer’s study would be significantly reduced: even if his claims had discouraged some scientists from pursuing the bacterial hypothesis, they’d be the final nail in the coffin of an already dying theory, rather than the main cause of its abandonment.

Second, explaining the PUD episode in terms of a wide dissemination of Palmer’s results would be undermined. As a result, this case would fail to serve as an example of how a high degree of information flow among scientists can lead to inefficient inquiry. While this point originates in Zollman’s work on network epistemology, it has become widely adopted across the philosophical literature, beyond discussions on formal models of science (e.g. Wray, 2010; Douven & Kelp, 2011; Nunn, 2012; Vickers, 2020; Peters, 2020; Killin & Pain, 2021). Since examples of harmful effects of a dense communication flow are hard to find, PUD has been particularly valuable as an illustration of this phenomenon. Hence, if our hypothesis is confirmed, philosophers need to find other suitable historical case studies in order to illustrate such a socio-epistemic mechanism. But as we shall argue in Sect. 4, this may be challenging.

Finally, if it turns out that there was hardly any research on the bacterial theory already in the 1940s, then the above question—why was the bacterial hypothesis abandoned in the 1950s?—wouldn’t be puzzling anymore. Instead, we would be confronted with different questions, raised by alternative possible histories of this case. One such possibility is that the bacterial hypothesis used to be popular at the end of the nineteenth century, after which it went through a gradual decline. Another option is that its decline wasn’t gradual, but abrupt and triggered at some point between the end of the nineteenth century and early 1940s. Finally, it is also possible that the bacterial theory had not been popular at any point in the first half of the twentieth century, but had always been a fringe research line. While previous historical discussions of this case had been based solely on a qualitative analysis, new quantitative methods, based on digital tools, could be fruitfully used to acquire new evidence and reveal which of the above scenarios is best corroborated. As we will argue, such new scenarios come with specific philosophical puzzles, which have so far not been considered.

Here is how we will proceed. In Sect. 2 we give a historical overview of this case-study focusing on the question, which factors might have contributed to the abandonment of the bacterial research program. In Sect. 3 we introduce the method of digital textual analysis, which we use to examine the historical claim that the bacterial research program was largely abandoned prior to the publication of Palmer’s study. In Sect. 4 we examine the consequences of our results for philosophical discussions of this episode, with a special focus on the literature in network epistemology. Section 5 concludes the paper.

## Etiological theories of PUD

In this section we provide a historical overview of the English-language research on PUD in the first half of the twentieth century, focusing on the question as for which factors, besides Palmer’s paper, could have indirectly contributed to the abandonment of the bacterial hypothesis of PUD. To this end, we will primarily rely on secondary sources from history of medicine^2^ and first-hand testimonies from gastroenterologists who were working on PUD during the period of our interest, in Britain (Christie and Tansey, 2002) and worldwide (Warren, 2005). These primary sources give an insight into personal factors that led researchers away from the correct hypothesis.

Before turning to factors that are relevant in considering the downfall of the bacterial research program (or the ‘germ theory of PUD’), we give a brief overview of different etiological theories of this disease researched in the first half of the twentieth century.

### Theoretical diversity

According to the received view that we engage with (Kidd and Modlin, 1998; Zollman, 2010; Pollock, 2014), two influential hypotheses of what causes PUD developed early on: on the one hand, the so-called acidity hypothesis, according to which the ulcer is caused by gastric juice corroding the stomach, and on the other hand, the bacterial hypothesis, which postulated bacteria as the cause of the disease. Eventually, the story goes, the latter strand of research was brought down by the paper by Palmer (1954) thus “setting back gastric bacterial research by a further 30 years” (Kidd and Modlin, 1998, p. 10). Our aim in this section is to show that, contrary to the commonly told story, the question of what causes PUD rarely took shape of a simple choice between the bacterial and the acidity theory.

While the research on PUD draws its origins from the late 16th century, modern gastroenterological study of the disease started in the 19th century. Moving on to the first half of the twentieth century, it is easy to notice a range of insufficiently corroborated etiological theories forming this research landscape (Miller, 2010, p. 105). For instance, Pollock (2014, Chapter 3) distinguishes eight different factors that were at some point considered important in the genesis of peptic ulcers. These include not only germs and the acid, but also factors related to anatomical pathologies, inborn predispositions (such as e.g. an “ulcer personality type”, see also (Miller, 2010, p. 102)), or psychological factors, such as stress (see also Jones, 2012, p. 13). What is more, gastroenterology was slow to develop as a specialized field, partly because there was no general agreement among surgeons and physicians on how to best treat digestive diseases (Miller, 2010, p. 105). Notably, however, the treatment of PUD–whether pharmacological or surgical—was mainly focused on reducing the acidity in the stomach (Miller, 2010, p. 105).

The research in the 1940s and the 1950s witnessed an increasing focus on the role of physiological and psychological factors on the development of PUD. *The Lancet* editorial from the end of the 1940s nicely illustrates this point: it posits that theories of peptic ulceration inevitably center around two possibilities: heightened erosive potency of gastric contents, or lowered anti-acid resistance (“Ætiology of Peptic Ulcer. Editorial”, 1949, p. 997). At the similar time, a number of editorials from *The American Journal of Digestive Diseases*^3^ emphasized in turn the psychological causes of PUD, such as anxiety and stress. The appearance of an ulcer was considered to indicate a reduced capability of the body to prevent ulcers, rather than a result of increased external ulcerogenic factors (such as bacteria).

Altogether, the research on PUD shifted away from a mono-causal and towards multi-causal approaches, and away from acidity as the sole etiological factor and towards the overall physiological balance in the stomach, including the failure of its anti-ulcer mechanism (Connell, 1949). This reflects the overall trend in medicine at the time. While in the beginning of the 20th century the medical research was largely driven by a mono-causal perspective, closely related to the germ paradigm of disease (originating in the works of Koch and Pasteur), the situation started to change around the 1950s with the emergence of the chronic disease epidemiology (Carter, 2003; Šešelja & Straßer, 2014). In case of the research on PUD, the multi-causal perspective was already present prior to Palmer’s study.

Some of the earliest indications that the idea of multiple causes was on the table comes from J. Shelton Horsley who commented that an ulcer may be produced by a combination of three factors: hyperacidity, toxic influences (possibly bacterial in nature), and the neurogenic (psychological) factors (Dragstedt, 1935, p. 579). After WWII, the popularity of multi-causal theories increased. For instance, according to Kirsner and Palmer, (1952, p. 615), “acid is indispensable” as a factor but “apparently not the only one”. In a similar vein, Sullivan and McKell, (1950, p. 14–20) introduced a ‘Theory of Multiple Etiology’, taking a form of a simple mathematical ratio, where the ulcer was a result of imbalance in the ratio of the sum of contributing factors, e.g. personality, precipitating emotional situations, genetic factors, etc., and the overall resistance to ulcers. Relatively strong ulcer-inducing factors, or relatively weak resistance, could both lead to ulceration.^4^ Remarkably, while the presence of acid was deemed essential, the bacteria were not mentioned by Sullivan and McKell. Taken together, the multi-causal approach meant that the etiological search space was more nuanced and complex than a simple choice between an acid theory and a germ theory.

However, the possibility that PUD was considered at the time as a multi-factorial disease is not discussed in the received view literature (e.g., Kidd & Modlin, 1998; Zollman, 2010). Even Pollock (2014), who discusses multiple etiological theories, portrays them as if they were pursued one at a time and treated as mono-causal accounts intended to be both sufficient and exhaustive. Thus, the evidence we provide above invites to reconsider the PUD case as that of scientists confronted not with a binary choice but with having to weight multiple factors in terms of their importance, perhaps in a way that the aforementioned theory of Sullivan and McKell from 1950 would suggest.

### Factors that played a role in the demise of the bacterial hypothesis

We now take a closer look at different issues, beside Palmer’s study, which could be explanatory of why the bacterial research program lost its popularity in the mid-twentieth century.

*The role of hyperacidity* As mentioned above, despite prolific arguments and the lack of agreement about the role of acid in ulceration, the acid theory seemed at the time to be the most fruitful hypothesis in terms of possible treatment (Christie & Tansey, 2002, p. 20). Therefore, the primary focus for treatment centered on regulating gastric secretion, which was reflected in a widely popular dictum: ‘no acid, no ulcer’, coined by Karl Schwarz in 1910 (Bralow et al., 1950).

The significance of acid as an etiological factor was in big part due to the work of Dragstedt (e.g. 1935) who demonstrated that a high degree of acidity in the stomach was alone capable of causing ulcers. This immediately led some scientists to consider hyper-acidity as the most immediate cause of ulcer (e.g. Rowland, 1937). As Pollock (2014, p. 93) comments, despite the lack of unanimity in the community, hyper-acidity became the main working hypothesis and the efforts towards an effective treatment were largely based on this assumption.

*Vagotomy* Another factor that played an important role in the decline of the bacterial research program is the success of a surgical procedure known as vagotomy. In order to treat ulcers, Dragstedt and Owens (1943) introduced a surgical method of cutting the vagus nerve, responsible for the acid secretion. Dragstedt established the viability of this procedure through a series of papers (Dragstedt, 1945; Dragstedt & Schafer, 1945; Dragstedt et al., 1947, 1949). Vagotomy appeared to work and until late 1970s it remained the most effective and reliable treatment for the condition, with comparatively fewest side-effects (Hobsley, 1994).^5^

*Problems in early bacteriological research* Since the introduction of Koch’s principles^6^ the major challenge for bacteriological theories was finding and identifying the disease-causing organism. Even though bacteria isolated from stomach ulcers were microscopically identified as early as in 1875, it was not clear which of them could play a part in the genesis of ulcers (Pollock, 2014, p. 85). Moreover, their reported frequency of occurrence in ulcerated stomachs was considerably lower than in other animals (Warren, 2005, p. 19). In the early 20th century Turck (1907, 1908) examined the link between Bacillus coli and PUD, but his findings were not successfully reproduced (Kidd & Modlin, 1998, p. 8). Soon thereafter, another researcher, Edward Rosenow, hypothesized that a different strand, Streptococci, was “commonly the original cause” of PUD (Rosenow & Sanford, 1915, p. 226) and attempted to induce ulcers with the aid of bacteria. Rosenow’s findings were influential and well-known (Pollock, 2014, p. 86) but later researchers again could not replicate the results using Rosenow’s technique (as reported in Ivy et al., 1950, p. 271). Thus, we can see that the initial studies, despite being based on the germ theory, posited wrong candidate microbes as etiological agents and as a result were not successfully replicated.

What’s more, while Rosenow believed in the etiological role of bacteria in ulceration, he held that it was the bacteria in and around the mouth and away from the abdomen that were to blame. In short, he looked for PUD-related bacteria outside of the stomach. This view was a particular expression of a “focal infection” theory, which posited that local sepsis in the teeth, tonsils, or sinuses, allowed a blood-borne spread of bacteria or toxins to other bodily areas, causing various diseases (Pollock, 2014, p. 89–98). As a treatment, Rosenow advised the surgical removal of the “loci of infection” (Rosenow, 1916, p. 359). However, the focal infection theory kept on drawing increasing criticism. It soon became evident that it is both life-threatening and practically impossible to try to remove all the loci of infection, and that one can have focal sepsis and still lead a perfectly healthy life (Pollock, 2014, p. 92). Eventually, by 1940 Rosenow’s theory was flatly rejected by Grossman (1940). Because of a misconceived mechanism for infection and unviable treatment, this strand of bacterial research faded away well before Palmer’s study.

Altogether, the significance of microbes in the stomach was not appreciated (Pollock, 2014, p. 89). Contemporary researchers regarded bacterial presence as “accidental” or at best secondary, following the ulceration but not causing it (Dragstedt, 1917; Smithies, 1935; Hinton, 1936; Winkelstein, 1936; Henry, 1942). This pattern continued outside of the US, as the presence of bacteria in the stomach kept being reported after the war (Barber and Franklin, 1946; Cregan et al., 1953) and even after Palmer’s paper (Bishop and Anderson, 1960; Franklin and Skoryna, 1966). Nevertheless, in each case the researchers did not assign any etiological role to the found microorganisms and maintained their beliefs that healthy stomachs are sterile.

An exception was the research by Freedberg and Barron (1940), who identified spiral bacteria in patients suffering from PUD. However, their study was small in scale and the results inconclusive. While their findings (subsequently cited by Palmer) provided some argumentative support to the bacterial research program, hardly anyone engaged in its pursuit.^7^

*Psychogenic Factors* Finally, the idea that gastric problems were in some way related to mental activity was a dominant theme in the North American and British literature on indigestion for centuries (Miller, 2010, footnote 30). This conjecture had a fertile ground to grow at the beginning of the 19th century, which marked the shift in medicinal practice towards a holistic approach, taking into account not only physical symptoms, but also the psyche, emotions and social environment of a patient (Spiro, 1998, p. 645; Miller, 2011, Ch. 5). The role of psychogenic factors was further corroborated by emerging physiological evidence linking brain malfunction and stomach disturbances (Miller, 2010, p. 101).

Another important development during this time was the rise in influence of Franz Alexander who in 1934 offered a psychogenic hypothesis of ulcer (Spiro, 1998, p. 645; Miller, 2010, p. 101). According to Alexander (1934), ulcer was developed as the result of suppressed subconscious tendencies, such as a desire to be fed, which in turn would trigger a negative somatic response leading to a disease. Furthermore, Robinson (1935) argued that PUD was found only among slender people of white race who as a result of their body type were supposed to have a disposition for mental instability, thus being at risk of developing ulcer. Inspired by these ideas, Davies and Wilson (1937) proposed the existence of an “ulcer type” of a person. Their work became highly influential and started a quest to define the “peptic ulcer personality” (Miller, 2011, p. 111–113). As Davey Smith (2005) argues, it was the belief in the ulcer-inducing power of stress that shifted the attention away from bacteriological research:

[T]he stress model served to block people from building on this [bacterial theory] and moving towards an answer ... Things may appear clear with hindsight, but people really were directed away from a treatment for peptic ulcers that worked—antibiotics—to ones that did not.

Coincidentally, the outbreak of WWII also boosted the influence of the psychogenic theory (Christie and Tansey, 2002, p. i). The incidence of peptic ulcer grew at an unprecedented rate, especially among troops internationally, and stomach disorders quickly became a major health complaint (Miller, 2010, p. 97). The war and the ulcers were associated so strongly that already early into the war, British practitioners began calling PUD a ‘military dyspepsia’ or a ‘war ulcer’. This novel rate of increase in ulcers was a new phenomenon and defied any logic in medical thinking. First, it contrasted with WWI, during which abdominal problems went relatively unnoticed (Miller, 2010, p. 97). Secondly, on the Eastern Front, few soldiers on the front-line developed ulcers, as opposed to those further back in the supply line (Miller, 2010, p. 97). Some researchers associated peptic ulcers with poor nutrition in the war-zone (Hoelzel, 1943; Steele 1944), but even as diet improved, the rate of occurrence kept increasing, reaching its peak in the mid-1950s (Jones, 2012, p. 1). As a result, in these post-Freudian days of the 1950s the psychosomatic factors, especially stress, in combination with ‘ulcer type personality’, were widely thought to be the main cause of the ulcer (Christie & Tansey, 2002, p. i). Looking for a connection between the army service and PUD continued in the US after the war and became a focus of several studies (Garbat, 1946; Halsted & Weinberg, 1946; Barrett, 1953; Palmer & Sullivan, 1952).

***

In this section we have provided an overview of developments other than Palmer’s paper, which contributed to the demise of the bacterial theory of the PUD etiology. This summary aimed to be primarily descriptive (rather than normative): while we presented a number of potentially relevant factors in the abandonment of the bacterial theory, we did not evaluate whether such a neglect was epistemically warranted (we will come back to this point in Sect. 4). Moreover, we do not claim we have established a definite answer as to what put the germ theory to a pause. However, we hope to have shown that there was a variety of factors that worked against it. In the next section, we will put forward a thesis that by the time Palmer’s infamous study was published, the germ theory had already been marginalized and cast aside by the overwhelming majority of scientists.

## The status of bacterial research program prior to Palmer’s study: digital textual analysis

In this section we examine the following historical question: to what extent was the bacterial hypothesis of PUD pursued prior to the publication of Palmer’s 1954 study? By answering this question we will be in a better position to judge the significance of Palmer’s result on the abandonment of the bacterial hypothesis.

The motivation for asking this question comes from a few separate considerations. First, as we have seen in the previous section, towards the 1950s, the overall research climate was not very forthcoming to the bacterial hypothesis. Second, assuming that the bacterial research program was active in the early 1950s, it is surprising that nobody noticed the methodological error underlying Palmer’s results. Finally, looking at the articles on the etiology of PUD published in the early 1950s, one can easily encounter articles that do not even mention bacteria as a potentially relevant factor (as noted by Šešelja and Straßer (2014)). Nevertheless, these indicators are insufficient evidential basis for answering the above query, whether Palmer’s paper was indeed a game-changer to PUD researchers. To approach the issue more systematically we turn to digital textual analysis of the relevant literature.

### Methodology

To address the above line of historical inquiry, we have performed a digital textual analysis of a selection of English language articles published in the period from 1943-1953^8^. More precisely, we have selected articles in PubMED database that have a MeSH Major Topic “Peptic Ulcer” and a a MeSH Qualifier “etiology”.^9^ Together, the Major Topic and the Qualifier yield a combined search term “Peptic Ulcer/etiology”, which we assumed to be sufficient for picking out the articles that are most likely to feature any significant research on bacteria as an etiological factor in PUD. Our complete search command was:

$$\begin{aligned} \begin{array}{c} {\text{``Peptic Ulcer/etiology''}}{[{\text{MAJR}}] AND}\\ ((\text{``1943/01/01''} {[{\text{PDAT}}]}: \text{``1953/12/31''}{[{\text{PDAT}}]}) AND {{\text{ English[lang]}}}) \end{array} \end{aligned}$$

Our search resulted in 186 hits, but actually consisted of 184 unique and complete papers, out of which we have managed to access 163 manuscripts. One of the papers was mistagged and was therefore removed from the bibliography.^10^ It is also worth mentioning that MeSH terms are either assigned to articles by human reviewers or automatically using natural language processing methods. In our case 80 out of 186 positions have been indexed automatically (without human supervision), making it not implausible that some “germ theory” articles were omitted.

To better understand this output, we will now elaborate on the status of PUD articles in the PubMed database in this time period.

For the period 1943–1953, PubMed lists 172,719 articles belonging to “Diseases Category”. Roughly 10.7% of these (18,477) are articles concerning “Digestive System Diseases”. In comparison, the “Infections” Major Term yields 45,221 articles (26.1%) and “Nervous System Diseases” yields 23,213 articles (13.4%). Within the “Digestive System Diseases”, PUD articles comprise roughly 14% (2579/18,477) making it roughly 1.5% (2659/172,719) of the more encompassing “Diseases Category”. Thus, PUD research appears to be a considerable area of study in this time period. Our selection of manuscripts is narrowed down to those that revolve around PUD’s etiology. Out of these, a substantial amount of articles comes from well-known specialised gastroenterological journals. For instance, there are 16 (out of 184) publications from *Gastroenterology*—American Gastroenterology Association’s (AGA) flagship journal and 12 from the *American Journal of Digestive Diseases*—also once the AGA’s flagship journal. Over a dozen of articles comes from non-specialised but equally well-renowned medical journals. For instance, 8 from the *Journal of American Medical Association*, 4 from The New England Journal of Medicine, 8 articles from the *British Medical Journal*, and 3, resp. 2 articles from the British journals *The Practitioner* and *Lancet*. Overall, our search results are representative exclusively of the English-language publishing in the period 1943–1953 given that they consist mostly of publications from the US (132 out of 184) with the rest of the articles in English from Europe and Southern Asia.

All the manuscripts have been digitally processed via the Optical Character Recognition software (OCR).^11^ To determine the presence of the bacterial research program in this body of manuscripts, we have examined the of occurrences of the following strings: ‘bacter’ and ‘spiroch’ (thereby identifying all the words that include the given string, such as bacteria/spirochetes.^12^) To digitally analyze the text in this way we have used *pdfgrep*, an open source Linux command line tool for searching text in PDF files (see https://pdfgrep.org/, accessed on July 1, 2021). More precisely, we have used the following command: pdfgrep -R -c “string”, which displays the number of instances of the given string in each file within the given folder. For each occurrence of the string, we have first-hand examined the context in which the string appears in order to determine whether the term is related to the bacterial hypothesis of PUD. In addition, for the sake of comparison, we have searched for the number of occurrences of the strings related to keywords of the acidity hypothesis, such as ‘acid’.

### Results

Among the analyzed manuscripts, we have found hardly any occurrence of the string ‘bacter’, and no occurrence of the string ’spiroch’. Out of 162 analyzed papers, only four mention bacteria as a possible cause of PUD. Out of these four papers, only one mentions bacteria in a more detailed context (Barber & Franklin, 1946), while the remaining three list it as one of numerous possible etiological factors (see Table 1). In contrast, string ‘acid’ appears in 145 of the analyzed manuscripts.

**Table 1:** Articles extracted via our search, which mention bacteria as an etiological factor in PUD

Article	The context in which bacteria are mentioned
Barber and Franklin (1946)	Bacterial hypothesis is taken seriously and previous studies mentioned; the main purpose of the study is determining the presence of bacteria in the stomach and duodenum at the time of operation
Lust (1952)	A book review: bacteria (from food and pharmaceuticals) are mentioned as one of the causes of mucosal damages causing gastro-duodenitis, which in turn causes PUD; this inflammatory process is considered unrelated to the secretion of the stomach
Mears (1953)	Bacteria are mentioned as one of nine possible etiological factors of PUD, discussed by a previous study
Arends (1951)	Bacterial infection mentioned as one of the many possible “extrinsic factors” that has been investigated in the context of PUD

The average occurrence of string ‘bacter’ in the whole set of examined articles is 0.41 times per article, while the average occurrence of string ‘acid’ is 14.58 times per article.^13^ Such a low average of bacteria-related strings, coupled with roughly a 30-fold disparity in the frequency of occurrence, is indicative of a largely abandoned status of the germ research program.

### Discussion

These results suggest that the bacterial hypothesis was indeed largely abandoned already before the publication of Palmer’s study, at least in the gastroenterological journal literature in English language.

We have further corroborated these findings by conducting an additional search in PubMed. Instead of focusing our search on the above mentioned corpus of articles that include the “Peptic ulcer/etiology” qualifier, we have searched PubMed for the same time period as well as the following decade based on ‘text words’,^14^ displayed in Table 2. The search command in this case had the following format:

$$\begin{aligned} \begin{array}{c} \hbox{``Peptic Ulcer''[TW]} AND {} \hbox{ ``string''[TW]} AND\\ ((\hbox {``1943/01/01''[PDAT]} : \hbox{``1953/12/31'' [PDAT])} AND \hbox{ English[lang])} \end{array} \end{aligned}$$

where string stands for the additional search term listed in Table 2.^15^

**Table 2:** The number of articles resulting from the search in the whole PubMed database for the given time periods, for publications in English language based on the given text words. The search terms were chosen at our discretion but we tried to minimize the author bias by including multiple diverse terms. The results for each search do not exclude the remaining strings, and hence, the same paper may be counted towards different search results

Search terms	1943–1953	1954–1964
‘peptic ulcer’ AND ‘bacter*’	10	13
‘peptic ulcer’ AND ‘spiroch*’	0	0
‘peptic ulcer’ AND ‘bacil*’	0	0
‘peptic ulcer’ AND ‘antibiotic*’	2	8
‘peptic ulcer’ AND ‘urea*’	2	4
‘peptic ulcer’ AND ‘pepsin*’	13	46
‘peptic ulcer’ AND ‘acid*’	71	136
‘peptic ulcer’ AND ‘vagus’	133	46
‘peptic ulcer’ AND ‘vagotomy’	234	205
‘peptic ulcer’ AND ‘therap*’	543	530
‘peptic ulcer’ AND ‘surg*’	645	730
‘peptic ulcer’	2579	3251

The number of hits for the acidity research program (‘acid*’, ‘vagus’, ‘vagotomy’) is again much higher than the number of hits for the bacterial research program. Moreover, the majority of the 10 articles resulting from the search for ‘bacter*’ do not belong to the bacterial research program (e.g. some are related to infections following a perforated ulcer, bacterial diseases that are complicated by the appearance of peptic ulcers, or the reduction of acidity in the stomach via substances of bacterial origin.) We list the number of hits for ‘peptic ulcer’ alone mainly to show the overall number of papers in this research area at the time (for the comparison with other articles in PubMed on digestive diseases see Sect. 3.1).

It is also worth mentioning that the number of articles on peptic ulcer available in the database rapidly increases towards the 1950s: out of 2659 hits for ‘peptic ulcer’ more than half are from 1950–1953. This is due to a more general trend in the PubMed database, which includes less than 10,000 articles published 1943–1944, compared to 250,000 in 1945–1949, and ca. 400,000 in 1950–1953.^16^

Finally, let us indicate some limitations of our study. First, one may wonder why we haven’t used citation analysis to examine the extent to which Palmer’s results had been cited at the time. The main reason for this is that the bibliometric data for the period between 1950-1970 is rather sparse. Hence, obtaining reliable information on how many scientists cited Palmer’s paper proved difficult.

Second, our study focused on a specific corpus of the relevant literature in gastroenterology, that is, English language literature on peptic ulcer indexed in PubMed in the period 1943-1953. Future studies may be extended to non-English language sources and further databases and archives. Moreover, looking into other historical sources may bring additional valuable insights into this episode. For instance, it would be interesting to examine funding applications at this time period and check whether those based on the bacterial hypothesis were submitted at the time, whether they were successful, etc.

## What can philosophers learn from this case-study?

As mentioned in the previous section, our results provide evidence for the claim that bacterial research program was largely abandoned already before 1954, the year when Palmer published his paper. Hence, it is not surprising that the bacterial hypothesis wasn’t investigated after Palmer’s publication: its pursuit had already been inactive for a whole decade. This is also why it is unlikely that the bacterial program was dropped *because of* Palmer’s study. If anything, the latter may have just assured scientists that the contemporary research community did not miss much by abandoning this line of inquiry.

However, the above conclusion opens a new set of problems and questions. In this section we list some of them, hoping to restart discussion on this historical episode and its role in the philosophical literature.

### Lessons for network epistemology

We first consider the implications of our results for previous employments of this case study in philosophical discussions. Our findings suggest that the given historical narrative, commonly used by philosophers, is unfounded. In particular, the claim alleging Palmer’s role in the premature loss of bacterial hypothesis seems insufficiently supported by historical evidence. However, it is precisely due to Palmer’s role that the PUD case has become one of the most common examples of the tension between the individual and group rationality used by philosophers of science. In particular, as mentioned in Sect. 1, PUD has been a central case study in the literature on network epistemology, illustrating how erroneous results obtained by one scientist can spread throughout the given scientific community, swaying it to a wrong theory. For instance, according to Zollman (2010):

In hindsight, Palmer’s study was too influential. Had it not been as widely read or been as convincing to so many people, perhaps the bacterial theory would have won out sooner. It was the widespread acceptance of Palmer’s result which led to the premature abandonment of the diversity in scientific effort present a few years earlier. (p. 21)

More recently, in reference to Zollman’s work O’Connor and Weatherall (2020) write:

Palmer’s findings were misleading. But they were so influential, that an entire generation of scientists turned away from the bacterial theory of ulcers and focused on treatments for stomach acid. (p. 40)

Our results reveal that such a narrative, rooted in Warren and Marshall’s interpretation of this historical episode, may not be accurate after all. If our findings are correct, the bacterial hypothesis had been largely abandoned already before Palmer’s study, in which case this episode cannot be used as an example of a scenario in which a quick spread of misleading information sidetracks the entire research community.^17^

But why should network epistemologists care about this? After all, they could simply use a different example to illustrate the same point. The problem is, however, that such examples may be rather hard to find. To see why this is the case, note that episodes illustrating the above claim that a high degree of interaction among scientists may lead to a premature abandonment of a fruitful scientific theory, have to satisfy two criteria: a) they should include a scenario in which the given scientific community initially pursues, but then abandons a hypothesis, which is in fact superior to its alternatives; b) such an abandonment should be primarily based on a wide-spread information flow of misleading results (rather than some other factors, such as dogamtism, various kinds of biases, etc.). Altogether, such cases would illustrate that a high degree of interaction among scientists can trigger a premature reduction in ‘exploration’ of different hypotheses, which is replaced by ‘exploitation’ of one of the sub-optimal ones.

Looking at the episodes of prematurely abandoned or ignored hypotheses (criterion a), such cases are already quite rare (the most prominent examples include Mendelian genetics, Wegener’s hypothesis of continental drift and the bacterial hypothesis of PUD). The main reason for this is that in most cases of a premature hypothesis rejection, the given scientific community remains split on the given issue, which then results in a scientific controversy rather than a widely adopted rejection of what is, in fact, a superior theory. Out of the above examples, only PUD has so far appeared to be a suitable case satisfying condition b) as an episode in which the abandonment occurred due to a wide dissemination of misleading results (rather than due to, for instance, dogmatic views of the involved scientists). But if, as we argue, this case does not fit the bill, we are left to wonder what other historical episode could be used as a replacement. After all, any suitable case would have to be such that a high degree of interaction, rather than some other factors, is causally relevant for the development of the given episode.^18^ The upshot is that the PUD case has seemed to be the only suitable candidate of this particular phenomenon modelled in the network epistemological literature, but the novel evidence we provide suggests it cannot play this role.

But couldn’t we still use the received narrative on PUD as a *plausible* historical scenario (even if inaccurate)? The problem with this idea is that the received narrative is not that plausible. If we assume that Palmer’s erroneous study was widely shared across the scientific community, it seems unlikely that nobody noticed a problem with it. In other words: a wide dissemination of erroneous results doesn’t simply increase the chance of a wide adoption of the given idea, but also of its critical assessment.^19^ It is also unlikely that Palmer’s results would trigger an outright rejection of the bacterial hypothesis, as maintained by the received view, rather than a controversy (which would preserve a theoretical diversity), as it usually happens in such cases.

Nevertheless, using highly idealized models to explain concrete historical episodes is not their only epistemic function. They can also have an exploratory function by providing a proof of possibility of a certain theoretical phenomenon or novel hypotheses about socio-epistemic mechanisms that underlie scientific inquiry (Šešelja, 2021). Even if we fail to empirically observe a causal mechanism that has been identified via an idealized model, this alone does not mean the given mechanism is philosophically uninteresting or irrelevant. On the one hand, the mechanism could remain empirically undetected for various reasons, including the possibility that some other empirical factors are typically more dominant, or that the phenomenon in question occurs only under very specific empirical conditions. On the other hand, the given mechanism could be theoretically relevant and explanatory of theoretical phenomena (such as scientific rationality taken in *abstracto*). In both cases the model could be motivated by a stylized scenario rather than a concrete historical one. However, this also means that the simulation cannot be considered validated in view of concrete historical episodes and arguably does not result from a properly integrated history and philosophy of science. Consequently, results of such simulations need to be taken with caution when drawing inferences about actual scientific inquiry. An argument that a certain phenomenon could be epistemically harmful because it has proved to be so in the past (where the model explains how and why), hasn’t been established.

Our study thus supports the claim that the results of the above mentioned network epistemological studies still need to be treated as exploratory. In particular, how significant the threat of a high degree of information flow among scientists is (e.g. for the purposes of science policy) remains an open question. While it may turn out that such a threat is indeed relevant under specific conditions of inquiry, which exact conditions these are (when interpreted in terms of actual scientific practice) has remained largely underspecified in the literature.^20^ From a more general point of view, our study provides support to recent calls for a modest treatment of results obtained by highly idealized agent-based models of scientific inquiry unless they have been empirically validated (Martini & Fernández Pinto, 2017; Frey & Šešelja, 2018; Thicke, 2020; Šešelja et al. 2020; Šešelja, 2021; for a somewhat different viewpoint see Mayo-Wilson & Zollman, 2021).

### Some open questions

As mentioned above, it was previously argued that the bacterial theory of PUD was worthy of pursuit in the 1950s, even after the publication of Palmer’s results (Šešelja & Straßer, 2014). As the authors point out, the bacterial research program not only had open lines of inquiry, but for each of the major objections directed against it (some of which have been elaborated in Sect. 2), there were clear methodological responses available at the time. Beside the objection coming from Palmer’s study, Šešelja and Straßer also examine the objection that the bacteria could not survive in the acid environment of the stomach, as well as the objections coming from the successes of the acidity research line. For instance, in response to Palmer’s results the research community had a counterargument coming from a study by Freedberg and Barron (1940), which emphasized the importance of using silver staining technique for detecting bacteria rather than hematoxylin-eosin stain, used by Palmer (see Footnote 7).

If we agree with this assessment, then the results presented in the current paper raise a number questions, both of historical and socio-epistemic nature. First of all, how come that a program which was worthy of pursuit in view of the arguments available at the time failed to be actually pursued? Was this just a result of an unfortunate division of labor, resulting from factors discussed in Sect. 2, or were some additional factors at play? This is particularly interesting in view of the claim by Fukuda et al. (2002) that prior to Palmer’s work there had been a consistent line of research on the bacterial origin of PUD throughout the first half of the twentieth century (see the quote in Sect. 1).^21^ Together with our findings, this would indicate that the bacterial research program declined over this time period. Such a course of events is interesting not only for discussions on the division of cognitive labor, but also for the problem of epistemic responsibility. For instance, we could ask: should anyone be held accountable for the abandonment of the bacterial research program? Answering this question is at the heart of contemporary discussions on collective epistemic responsibility and normative accounts of accountability of scientists as (unorganized) collectives (see e.g. Fleisher & Šešelja, 2021). Moreover, this problem is closely related to discussions on scientific pluralism as well (e.g. Longino, 2002; Chang, 2012) since the PUD case illustrates potential dangers of losing a fruitful line of inquiry.

Finally, the status of the bacterial hypothesis in non-English speaking literature is another open question worthy of further investigation, which may shed additional light on the overall dynamics of the medical community at the time.

## Conclusion

In this paper we have re-examined the history of the research on PUD, and the role of Palmer’s infamous study, which has long been considered to have played a central role in convincing other scientists that bacteria cannot be an etiological factor in this disease. To this end, we have used digital tools to systematically analyze a scope of journal articles published in English language in the decade before Palmer’s publication. Our results suggest that there had been hardly any active pursuit of the bacterial hypothesis already before Palmer’s publication. This indicates that the impact of a single influential figure on the whole research program is perhaps overestimated in the received view.

Even for those who would rather proceed with caution and who consider our results as just a piece of the puzzle requiring further investigation, our study should at least make them pause. The obtained results indicate that, at the minimum, we need to re-examine the received narrative before we take it to be an accurate historical presentation of the PUD episode. This is all the more important given the lack of historical evidence corroborating the alleged role of Palmer’s work in the history of PUD, as well as the lack of attempts at using quantitative tools for systematic digital analysis of the literature on PUD published throughout the first half of the twentieth century.

We will close by highlighting the methodological relevance of our study. The availability of digital tools makes re-examinations of historical episodes discussed by philosophers of science timely and relevant. In addition to the method of textual analysis employed in this paper, other types of related methods may be even more suitable for similar investigations. In particular, citation analysis in view of bibliometric data may provide insights into social networks characteristic of the scientific community at the time.^22^ As we have mentioned, the reason we have turned to textual analysis rather than to the citation analysis is that the bibliometric data for this time period is rather sparse. Hence, obtaining reliable data (e.g. on how many scientists cited Palmer’s paper) proved difficult. However, for more recent case-studies, bibliometric data may be a valuable additional evidence.

## Footnotes

1 See also Kidd and Modlin (1998), Fukuda et al. (2002), Warren (2005, p. 18).

2 Sources include: two articles on the history of abdominal illness in Britain during WWII by professional historians of medicine (Miller, 2010; Jones, 2012); an MD thesis in the history of medicine by Pollock (2014), which comprises a chapter on the history of etiological theories until 1960s; a historical overview of ‘germ theory’ research until Palmer’s paper (Kidd and Modlin, 1998); a critique of the biopsychosocial model with PUD as a case study (Davey Smith, 2005). The authors of the last two publications are medical practitioners and were included to represent two different interpretations of factors leading to the abandonment of the ‘germ theory’. Moreover, we have included a more recent case study on this topic, written in the field of integrated history and philosophy of science (Šešelja and Straßer, 2014).

3 For example Cornell et al. (1944), “Editorial” (1954b), “Editorial” (1954a), and “Peptic ulcer and “ordinary” anxiety” (1950).

4 Sullivan and McKell summed their theory in the following formula: $u = \dfrac{a+b+c+d+x}{r}$, where *u* = ulcer, *a* = constitutional and genetic factors, *b* = personality, *c* = precipitating emotional situations, *d* = physical injury, *x* = unique factors, *r* = resistance.

5 This is not to say that vagotomy was harmless: it still had a significant mortality rate. For example, Edwards et al. (1963) report the operative mortality rate of 2.7 % (see also Šešelja and Straßer, 2014, p. 437).

6 In the second half of the 19th century Koch presented a set of postulates for accepting the etiological role of bacteria: (1) The organism must be shown to be constantly present in characteristic form and arrangement in the diseased tissue; (2) the organism must be isolated and grown in pure culture; (3) the cultured organism must be shown to initiate and reproduce the disease when reinoculated into a healthy body; (4) the organism must be re-isolated from the experimentally infected organism (Thagard, 2000, p. 59).

7 A particularly interesting aspect of Freedberg and Barron’s study is that they explicitly advise against the employment of hematoxylin-eosin staining technique (later on used by Palmer) for the identification of bacteria, since in contrast to silver staining (subsequently used by Warren and Marshall), it did not reveal the spiral bacteria. Note also that while Palmer cites their paper he does not comment on Freedberg and Barron’s warnings concerning the staining techniques (see also Šešelja & Straßer, 2014, Sect. 5).

8 The textual analysis did not consider books published during this period. These resources could be included in futures studies.

9 Medical Subject Headings (MeSH) Major Topic terms are assigned to articles by the U.S. National Library of Medicine. The MeSH Major Topics classify articles in terms of a disease, a type of an injury, or a pathological condition that a medical article focuses on, and they are usually obtained from the title and/or statement of purpose (see https://www.nlm.nih.gov/bsd/disted/meshtutorial/principlesofmedlinesubjectindexing/majortopics/ accessed on July 14, 2019). MeSH Qualifiers allow to bring together citations focusing on a particular aspect of a subject (which usually is a particular disease). The “etiology” Qualifier is “used with diseases for causative agents including microorganisms and includes environmental and social factors and personal habits as contributing factors”. The above qualifier includes, among others, the “pathogenesis” (see https://www.nlm.nih.gov/mesh/qualifiers_scopenotes.html) and the “microbiology” Qualifiers (see https://www.nlm.nih.gov/mesh/subhierarchy.html).

10 The mistagged paper, unrelated to PUD, is Twiss and Carter (1952). A paper by Monro (1945) was published twice, while a paper by Chattopadhyaya (1951) was split in two. The main reason we couldn’t retrieve all the articles is that they are not available in libraries across Germany, which means that obtaining them would be significantly more costly.

11 More precisely, we used OCRmyPDF software based on Tesseract, an open source OCR engine, see https://ocrmypdf.readthedocs.io/en/latest/index.html. Our results are based on the 4.1.0 release of Tesseract, see https://github.com/tesseract-ocr (both links accessed on July 1, 2021).

12 Spirochetes are spiral bacteria discussed by Palmer and others (e.g. by Freedberg and Barron, see Sect. 2) in the context of PUD.

13 We have further validated these results by examining the occurrences of related terms, such as “microbe” and “germ”, which were very scarce and in no instance related to the etiological role in PUD. As a basic test we checked the occurrences of the string “ulcer” which indeed appeared in 100% of the articles.

14 Text words search includes “all words and numbers in the title, abstract, other abstract, MeSH terms, MeSH Subheadings, Publication Types, Substance Names, Personal Name as Subject, Corporate Author, Secondary Source, Comment/Correction Notes, and Other Terms $\dots$” (https://pubmed.ncbi.nlm.nih.gov/help/#tw, accessed on July 1, 2021).

15 In order to stay sufficiently broad in our search we included each decade plus an additional year.

16 This disparity could be due to multiple reasons: from the increase in the number of post-war publications to a higher coverage by the relevant indexes to medical periodical literature, which were for this time period selective (see https://www.nlm.nih.gov/databases/databases_oldmedline.html, accessed on July 1, 2021).

17 We are not suggesting that the scientific community at the time was not tightly connected, but rather, that factors other than the connectedness of the community and the structure of its information flow may be more explanatory of the loss of the bacterial hypothesis.

18 This may be challenging: for example, in case of the continental drift debate, biased outlooks of North American scientists played an important role in their rejection of Wegener’s hypothesis of continental drift, see Oreskes (1999), Šešelja & Weber (2012).

19 Think of Wakefield’s fraudulent study aiming to show an association between the measles-mumps-rubella vaccine and autism, which received primarily critical response from the scientific community (see e.g. Suelzer et al., 2019).

20 For some attempts at specifying such conditions by means of robustness analysis see Rosenstock et al. (2017), Frey and Šešelja (2020).

21 While their claim is based on 15 studies related to the bacterial hypothesis of PUD conducted between 1875 and 1940 (Fukuda et al. 2002, p. 18), it would be interesting to apply digital textual analysis to this time period as well and reexamine whether this was indeed the case, or whether the bacterial research program had been rather fringe all along.

22 For examples of using social network analysis in the context of integrated history and philosophy of science see Claveau and Herfeld (2018), Herfeld and Doehne (2018). For a more general discussion on digital approaches to philosophy of science see Pence and Ramsey (2018).
